# Sporotrichosis: A Review of a Neglected Disease in the Last 50 Years in Brazil

**DOI:** 10.3390/microorganisms10112152

**Published:** 2022-10-30

**Authors:** Carmen Magaly Alvarez, Manoel Marques Evangelista Oliveira, Regina Helena Pires

**Affiliations:** 1 Laboratory of Mycology and Environmental Diagnosis, Postgraduate Program in Health Promotion, University of Franca, Franca 14404-600, SP, Brazil; 2 Faculty of Veterinary Medicine, Universidad Agraria del Ecuador, Guayaquil 090104, Ecuador; 3 Laboratory of Taxonomy, Biochemistry and Bioprospecting of Fungi, Oswaldo Cruz Institute, Fiocruz, Rio de Janeiro 21040-900, RJ, Brazil

**Keywords:** sporotrichosis, zoonosis, *Sporothrix* spp., neglected disease

## Abstract

Sporotrichosis is caused by fungi belonging to the genus *Sporothrix*, which saprophytically are found in plants and organic matter. However, cats are highly susceptible to contamination with fungal spores and, when they become sick, they can transmit it to other animals and to man. The objective of this study is to carry out a systematic review on the emergency, diagnosis, clinical symptoms, therapeutics, and control of zoonotic sporotrichosis. Published data covering the last 50 years using a combination of keywords were selected to answer the question: Why has the zoonotic sporotrichosis been a neglected disease up to now? A total of 135 studies were included in this review. The studies emphasize that in recent decades, Brazil has experienced an unprecedented zoonotic outbreak of sporotrichosis. Advances on the genus *Sporothrix* allowed one to associate thermotolerance, capacity for melanin synthesis, potential for adhesion to tissue macromolecules, ergosterol peroxide production, and expression of virulence proteins as tools for infection and invasion in *S. brasiliensis*, the main species involved, although cases with *S. schenckii* or *S. lurei* were also reported. Correct diagnosis, early treatment, basic educational measures that emphasize responsible ownership of animals and reproductive control programs for felines can contribute to the control of zoonosis.

## 1. Introduction

Sporotrichosis is considered an implantation mycosis caused by species of the *Sporothrix* genus that usually affects the skin at the cutaneous and subcutaneous levels [1]. The main species *Sporothrix sckenckii* was described by Benjamin Schenck, in the United States, in 1898 [2]. In Brazil, Lutz and Splendore described, in 1907, the first cases of sporotrichosis in humans and rats [3]. In the classic form, infection occurs through traumatic inoculation of the fungus due to perforation by spines or wood chips, which evolve into ulcerative skin lesions, in exposed areas such as the extremities or interdigital areas [4]. Conidia can be inhaled, producing lung infections that are challenging to treat; it can affect bones and joints. Indeed, the disease clinical forms will depend on factors such as the individual’s immune status [5], and by environmental conditions that can trigger the expression of different fungal virulence factors [6].

Until the late 1970s, the disease was common in gardeners, farmers or people who had contact with plants and soil of natural environments where the fungus could be present in organic materials and therefore considered an occupational disease [1,7]. However, sporotrichosis can affect several species of mammalian animals [2,8]. Transmission by bites or scratches, mainly from cats (*Felis catus*), which can carry large amounts of yeast between their claws in addition to their close contact with humans, allowed the characterization of sporotrichosis as a zoonosis, mainly affecting families (with unfavorable infrastructure and socioeconomic conditions with sick cats at home) and veterinary professionals and assistants [1,9].

In recent years, studies using molecular techniques, allowing the identification of phylogenetically related species (criptic species) and new *Sporothrix* species, have been described [9,10,11,12,13,14,15,16,17], including *Sporothrix brasiliensis* which has been implicated as main responsible agent of zoonotic sporotrichosis in Brazil. However, the prolonged treatment of patients, even in the mild forms (cutaneous and lymphocutaneous), combined with the abandonment of the sick animals (mainly the cats) by the tutors due to the aspects of the cutaneous lesions, the difficult and prolonged therapeutic management and high cost, or fear of getting sick from the fungus, have contributed to the lack of disease control, mainly in metropolitan regions, with the occurrence of epidemics and outbreaks.

Furthermore, in Brazil, the recognition of sporotrichosis as an emerging and neglected disease with a relevant socioeconomic impact on the country’s development obstructs the identification of risk factors and groups and contributes to a precariousness of definition of relevant and timely prevention and control measures. In this context, this systematic review addresses advances in the knowledge of zoonotic sporotrichosis, focusing on Brazil, and discusses the points that contribute to sporotrichosis still being considered a neglected disease.

## 2. Materials and Methods

The review was guided by the Preferred Reporting Items for Systematic Reviews and Meta-Analyses (PRISMA) [18]. Two investigators (C.M.A. and R.H.P.) independently performed the searches from Feb 2022 to June 2022, using the descriptors in MEDlars onLINE (MEDLINE)/US National Library of Medicine (PubMed), The Latin American Caribbean Health Sciences Literature (LILAC), Virtual Health Library (VHL), ScienceDirect and Scopus databases. The descriptors used were: “Sporotrichosis, Fungal Infection, Pet, Zoonoses AND Brazil, Sporotrichosis AND Diagnosis AND Treatment” for searches in the English language; “Esporotricosis, Infecciones Fúngicas, Mascotas, Zoonosis AND Brasil, Esporotricosis AND Diagnóstico AND Tratamiento” for searches in the Spanish linguague; “Esporotricose, Infecção Fúngica, Animais de Estimação, Zoonoses AND Brasil, Esporotricose AND Diagnóstico AND Tratamento” were the terms in Portuguese for the searches. The period investigated covers the past 50 years (1971 to 2022) with a focus on the Brazilian endemic and the studies were selected according to eligibility and exclusion criteria (Table 1). A third researcher (M.M.E.O.) checked the quality of the selected studies.

## 3. Results and Discussion

**Figure 1:**
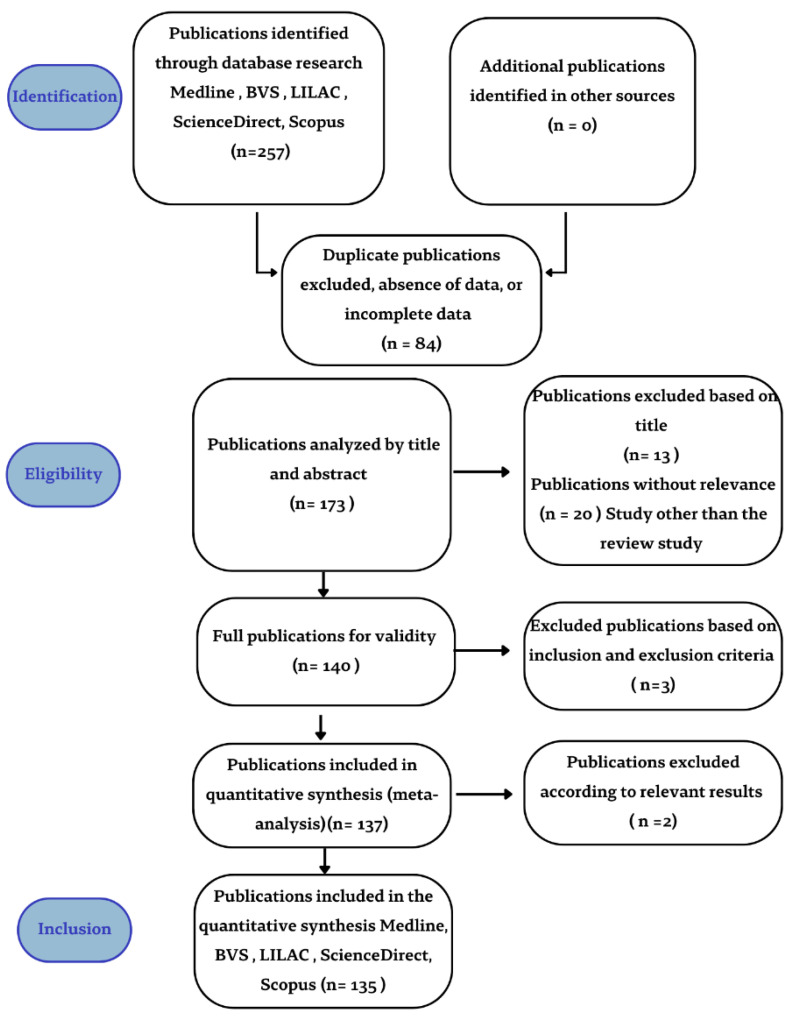
PRISMA flowchart of the literature systematic review.

A total of 257 studies was selected from the databases. Overall, 173 papers were screened through abstract and title reading after the removal of the duplicates and 140 studies were validated. After careful selection based on the inclusion and exclusion criteria, the studies were read in full, and 135 studies were chosen to compose this review. A PRISMA flow chart of the selection process and screening is provided in Figure 1.

### 3.1. Emergence of Sporotrichosis as a Zoonotic Disease

From the first notification of the fungus in 1898 until the next century, *S. schenckii* was considered a single fungal species that presented several degrees of pathogenicity, route of infection and clinical aspects [6,16]. Based on physiology and molecular analyses, *Sporothrix schenckii* was classified as a complex and was subdivided into: *S. brasiliensis*, *Sporothrix mexicana, Sporothrix globosa* and *S. schenckii* sensu strictu [10]. Later, *Sporothrix lurei* was incorporated into the complex [11]. Non-pathogenic species such as *Sporothrix albicans, Sporothrix pallida* and *Sporothrix nivea* were jointly named as *S. pallida* through phylogenetic analyzes [19,20]. Three other species, *Sporothrix stylites, Sporothrix humicola* and *Sporothrix lignivora*, were identified using a molecular approach [15]. DeBeer et al. [21] described 53 cryptic species, phylogenetically related into the *Sporothrix* genus. The species *S. brasiliensis, S. schenckii* sensu stricto, *S. globosa* and *S. pallida* complex have been the main species related to sapronotic transmission while the species *S. brasiliensis* and *S. schenckii* are related to both zoonotic transmission and horizontal animal transmission [22]. The species *S. brasiliensis* is a geophilic thermally dimorphic fungus with high virulence and evasive capacity of the immune response; it is transmitted by biting, scratching or contacting with the exudate of a cat’s skin lesions [23]. In addition, *S. humicola* are considered causal agents of feline sporotrichosis [24]. *S. luriei* was associated with human sporotrichosis in two human cases, in Africa [11] and India [25], respectively, and as an agent of animal sporotrichosis, was reported in a Brazilian canine case [26].

Due to the large number of cases, Brazil has been considered the epicenter of zoonotic transmission, especially cat-transmitted sporotrichosis. From 1997 to 2001, the first cases of zoonotic sporotrichosis were reported in the city of Rio de Janeiro and in adjacent municipalities (Duque de Caxias, Queimados and São João de Meriti), with 178 patients treated with epidemiological linkage with cats that had similar skin lesions [27]. Between 2002 and 2004, 572 patients were seen in the Rio de Janeiro metropolitan region [27,28,29]. The most frequent clinical presentation was the lymphocutaneous form, followed by the localized cutaneous form, affecting mainly the upper limbs, which are more exposed to scratches or bites during the care of sick felines [30]. Children, the elderly, and women with low socioeconomic status have been the most affected groups [28,29,31,32]. Another large epidemic outbreak was observed between the years 2005 and 2008 [33]. In most cases, the species *S. brasiliensis* was identified and both animal transmission (cat–cat and cat–dog) and zooonic transmission (cat–humans) were detected [34,35]. Approximately 5113 feline cases occurred from 1998 to 2018 and ≈ 5000 human cases from 1998 to 2020 [33,36]. Clinical manifestations in humans’ cases that are unrelated to sporotrichosis such as hypersensitivity reactions, spread of infection, nervous system tropism and ocular infections have been associated with *S. brasiliensis* [37,38]. So, new clinical presentations that were uncommon until the zoonotic epidemic were identified, and the specialists have proposed a new clinical presentations classification [39].

**Figure 2:**
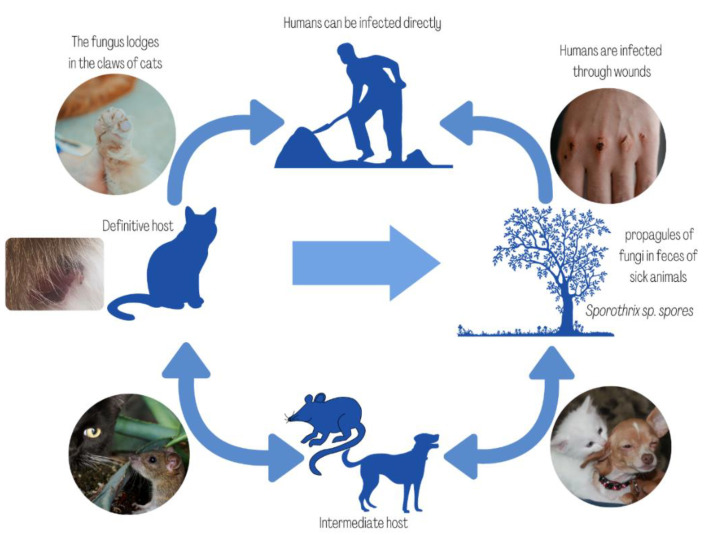
Evolutionary cycle of *Sporothrix* spp. infection.

The mandatory notification of cases to the health authorities was established in 2013, in the Rio de Janeiro state [40]. The notification was also implemented in some other Brazilian states such as Pernambuco, Minas Gerais and Paraíba and in the capital of the state of Bahia, the city of Salvador [41,42,43,44]. Late diagnosis, and especially late treatment, has led to the sporotrichosis progression, causing the disease to spread across 25 Brazilian states [36,45]. Indeed, the zoonotic transmission cases in Rio Grande do Sul and Paraná [46,47,48], Brazilian states bordering Argentina, has allowed the *S. brasiliensis* propagation across that country as reviewed by Etchecopaz et al. [49]. In Peru, cases of sporotrichosis have also been reported in areas of low socioeconomic conditions such as Cajamarca, Apurímac and Amazonas, and between 1991 and 2014, 94 patients were infected with *S. schenkii* [50]. Most were adult men involved in agricultural activities with a predominance of the cutaneous-lymphatic form followed by the localized cutaneous form, in both upper limbs [50]. In general, animal sporotrichosis cases reported in the period 2007–2021 predominate in South America followed by Asia and Europe, while North America and Africa showed the same number of cases and rare cases have been reported in Central America and Oceania as reviewed by Morgado et al. [24]. Although in the past, the zoonotic transmission of sporotrichosis was reported only as sporadic and self-limited cases, in Brazil [45], the poor sanitary conditions and the large population of cats in contact with humans which harbor many yeasts, contributed to the high zoonotic potential for *Sporothrix* transmission [8,51]. Furthermore, the epidemic experienced in recent years may be related to the complex adaptive evolutionary strategies of the fungus (Figure 2). For instance, phylogenetic analysis shows an increase in infection by *S. brasiliensis, S. schenckii* and *S. globosa* in vertebrate hosts [34,52] while genomic studies indicate a lower abundance of plant degrading enzymes in *S. brasiliensis* and *S. schenckii* [53]. Futhermore, the mycelium-to-yeast transition in *Sporothrix* varies between the clades that exhibit different pathogenic behaviors [34,54,55]. More virulent clades show greater efficiency in the mycelium-to-yeast transition, such as *S. brasiliensis*. Indeed, the conversion to the parasitic stage is not necessary when reaching the human host, since *S. brasiliensis* is already in the yeast form in the nails of cats [28]. Nevertheless, in *S. chiliensis* and *S. mexicana*, species of the environmental clade, with attenuated virulence, the morphological transition is more difficult [52]. Zoonotic transmission having as etiological agent *S. schenckii* s. str. and cats as carriers were also identified in Malaysia [56,57].

Therefore, the change in the pattern of transmissibility of the fungus, as well as the *S. brasiliensis* emergence as an agent of greater virulence, aggravating the cases in humans and animals in Brazil, points to the need for a deployment and implementation of surveillance and control of sporotrichosis by public agencies with a the concept of One Health, introduced in early 2000 by the World Health Organization (WHO) and the Regional Office of the WHO for the Americas—the Pan American Health Organization (PAHO).

### 3.2. Sporotrichosis Diagnosis

The correct identification of sporotrichosis agents is beneficial for epidemiological surveillance and for promoting effective public health policies. Among the classic laboratory procedures (Table 2) for the sporotrichosis diagnosis, the isolation in culture medium is the gold standard, which must be followed by macro and micromorphological identification, in addition to the in vitro thermoconversion test [45,46,58,59,60,61,62,63]. The ELISA (Enzyme Linked ImmunonoSorbent Assay) method, used for several years for the diagnosis of sporotrichosis, has been a useful tool for the sporotrichosis serological diagnosis [64,65,66,67], although some of these tests may cross-react with other fungal diseases [66]. Molecular diagnostic methods including conventional Polymerase Chain Reaction (PCR), Random Amplified Polymorphic DNA (RAPD), PCR-Restriction Fragment Length Polymorphism (PCR-RFLP), and gene sequencing (calmodulin, beta-tubulin, translation elongation factor-1-alpha (TEF1) and translation elongation factor-3 (TEF3) EF-1α) [9,10,11,13,14,15,20,21,32,52,53,56,68], or more recently, methods that employ the Matrix Assisted Laser Desorption Ionization Time-of-Flight Mass Spectrometry (MALDI-TOF MS) technique [69,70], have also been used for the diagnosis of sporotrichosis or for isolates genotyping (Table 3) more efficiently than other phenotypic diagnostic methods. Knowledge of the molecular profile of the fungus contributes to the understanding of the dynamics of occurrence of *Sporothrix* species, especially those with zoonotic involvement. Furthermore, these methods showed good sensitivity for identifying *Sporotrhix* from samples of spleen, liver, lung, heart, brain, kidney, and tail, as well as faeces from infected animals. From conidia, it also obtained great accuracy and effectiveness for the *Sporotrix* species identification [68].

Diagnosis at an early stage, especially in cats, after proper treatment, allows the cure. Nevertheless, fungal growth in culture may not be observed and there may be microbiological contamination, or this methodology would not be accessible for diagnosis. Classic methods of mycological identification are laborious and time consuming. In addition, they require considerable knowledge for the correct morphological identification of the fungal species [69,71]. Although molecular methods and the MALDI-TOF technique allow for the rapid and accurate identification from the clinical specimen or from environmental samples, the cost and technical demands associated with these methods make them inaccessible. The vast majority of feline or canine cases, when submitted to care, are performed in veterinary clinics that use only direct examination with KOH or Gram-stained imprints (Giensa staining is rare) for diagnosis. Currently, the startup BioInsumos e Diagnosticos (BiDiagnostics), with support from the Innovative Research Program in Small Businesses (PIPE) of the Foundation for Research Support of the State of São Paulo (FAPESP), developed a rapid serological test for the detection of sporotrichosis in domestic cats, based on the concept of Point of Care (PoC), which consists of an ELISA method with *Sporothrix* spp. antigen, allowing a quick and timely diagnosis in the veterinary consultation. This immunochromatographic test from a small blood sample obtained from the plantar pads of the animal detects the triagem of feline sporotrichosis, thus helping with safe and fast diagnostic tools.

### 3.3. Clinical Manifestations

Although sporotrichosis has a good prognosis, there is an increase in atypical clinical and disseminated forms requiring long periods of hospitalization and treatment, which has financially burdened the health system. These clinical forms have been observed in human patients infected with *S. sckenckii* and, mainly, in patients infected with *S. brasiliensis* due to zoonotic transmission mainly by cats, in endemic areas of Brazil such as the Southeast and South regions.

The zoonosis has been predominant in female patients, probably due to the high number of women working as housewives or cleaning women who are in closer contact with animals in the home [30,32,37]. In addition, a previous study showed that feces from sick animals can contaminate the soil with fungus propagules and contribute to the spread and persistence of the fungus in the environment [73]. Fixed clinical forms located in the upper limb followed by the lower limb and face are the clinical forms most seen in the patients from Brazilian endemic areas [32,74], possibly due to continuous exposure to fungal conidia, which could gradually confer immunity and prevent spread through the lymphatic system [75]. This clinical form consists of a single lesion, usually like the inoculation chancre, with no regional lymphatic spreading, and has also been reported in Mexican patients infected with *S. schenckii* residing in endemic areas of sporotrichosis [76]. An acute eruption of erythematous nodules on the lower limbs that may be accompanied by fever, malaise and arthralgia characterizes erythema nodosum. Both this clinical manifestation and erythema multiforme (vesiculobullous skin manifestations) may be associated with hypersensitivity reactions [77,78]. Cases of conjunctivitis, episcleritis, uveitis, choroiditis and granulomatous conjunctival lesions with hyperemia, secretion and edema have also been reported, although with low frequency, probably the result of traumatic injury or hematogenous dissemination [32,37,79,80,81,82]. The human host immune response to *Sporothrix* is due to both a cell-mediated immune response and a humoral immune response that produces antibodies against the fungal wall [55,83]. The spread of the disease is related to deficiencies in cellular immunity, as in the case of patients infected with the human immunodeficiency virus (HIV) [76], alcoholism, the chronic use of illicit drugs or the use of immunosuppressive medication [34,82]. The lungs can be affected either by inhalation of fungal propagules or by hematogenous spread. Signs and symptoms may include cough, dyspnea, hemoptysis, among others, and radiological images (chest radiography or computed tomography) show the upper lobes with cavitary aspects, reticulonodular infiltration, or even fibrosis or tumors [84,85]. Altogether, the different clinical forms of sporotrichosis contribute to erroneous diagnoses in endemic areas due to the lack of knowledge of physicians. Furthermore, injuries acquired in a traumatic way through scratching and biting by cats or by non-traumatic means such as coughing or sneezing from cats, direct contact with skin and animal secretions have characterized zoonotic sporotrichosis as the only infection caused in the yeast phase by endemic dimorphic fungi [86].

**Figure 3:**
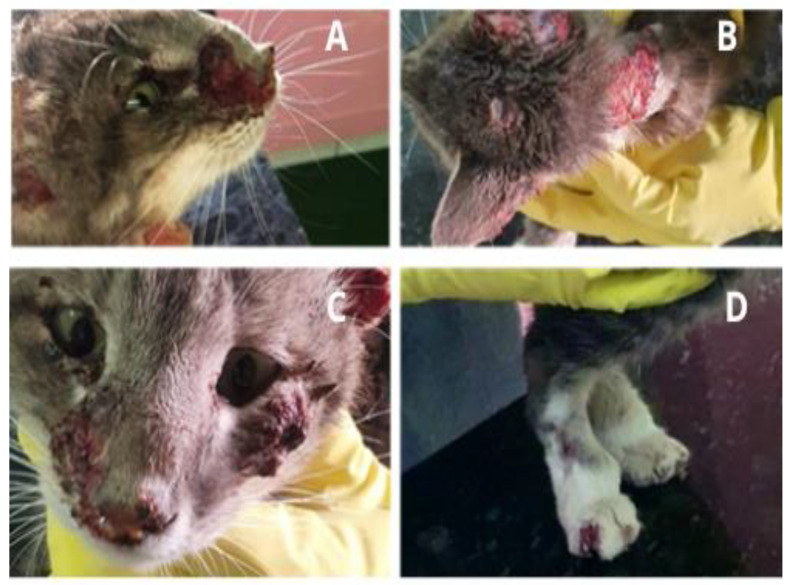
Clinical aspects of cutaneous sporotrichosis in cats. (**A**) Nasal region with crusted skin lesions on the nasal bridge. (**B**) Multiple skin lesions on the back and ears. (**C**) Crusted lesions on the cat’s muzzle and face (**D**) Paronychia on the forelimb. Images provided by Sabrina Manzoli, Franca, SP, 2020.

Feline sporotrichosis (Figure 3) has been described in some countries around the world, although Brazil has the highest number of identified cases [87]. The species *S. brasiliensis* has been identified in most cases, although *S. sckenckii, S.*, *S. humicola,* and possibly *S. chilensis* have also been reported [6,7,8,9,10,11,12,13,14,15,16,17,18,19,20,21,22,23,24,25,26,27,28,29,30,31,32,33,34,35,36,37,38,39,40,41,42,43,44,45,46,47,48,49,50,51,52,53,54,55,56,57,87,88,89,90,91,92,93]. The disease in cats is mainly reported in free-roaming intact males and, generally, the increase in the number of cases in cats is accompanied by an increase in the number of human cases in densely populated urban areas [32,45,48]. The lesions of feline sporotrichosis have ulcerative characteristics and are located in the head, thorax and extremities region (Figure 3), and may present as an isolated lesion or multiple skin lesions (cutaneous clinical form). These lesions usually also manifest themselves in the tutor with lymphangitic path and larger nodules, constant recurrences, and difficult healing, even with the use of antifungals. In some cases, these lesions may become necrotic, exposing underlying tissues and can cause deformities and putrid odor, which often gives rise to myiasis and subsequent death [16,94]. The systemic involvement and respiratory signs are frequent [95] and closely linked to the animal’s immunity and the existence or not of comorbidities such as feline immunodeficiency virus (FIV) or feline leukemia virus (FeLV) leading to disseminated forms of the disease [32,35,96]. In addition, these lesions (exudate tissues), feces and claws are rich in fungal propagules which efficiently contribute to the transmission of infection even in the absence of skin lesions [27,45,89].

Cytopathological examination has been the technique of choice for the presumptive diagnosis of feline sporotrichosis in veterinary clinics and small laboratories in most Brazilian cities due to its good sensitivity [63]. Sporadically, histopathology or serology are used. Polymerase chain reaction (PCR) and the MALDI-TOF technique have been used to detect *Sporothrix* infection in cats in large metropolitan centers or for research purposes [87]. This fact potentiates the infection of other animals and humans due the lack of early laboratory and clinical diagnosis of feline cases. In addition, *S. brasiliensis* is already in the yeast-like phase, colonizing the nail and oral cavity of cats, which favors human infection, since it is not necessary to convert to the parasitic phase when it reaches the human host [28,97]. Another relevant aspect is the ability of *S. brasiliensis* to form biofilms on cat claws, causing the persistence of the fungus and increasing the transmission capacity during traumatic inoculation [98].

Although canine sporotrichosis has been poorly described in Brazil, *S. brasiliensis* predominates in canine isolates [91,99,100,101]. *S. sckenckii* has also been reported in some studies [91,102]. *S. lurei* was also reported in only one case in the state of Rio Grande do Sul [26]. Some reports have not defined the species of *Sporothrix* [103]. The route of canine infection has been suggested through the environment or contact with infected cats through scratching and biting [104]. Skin lesions tend to be fewer in canine sporotrichosis with a predominance of the cutaneous form [102]. Schubach et al. [105] studied 44 dogs in the city of Rio de Janeiro and reported single lesions in 18 dogs, two to four lesions in 17 animals, and nine animals having five or more lesions. Twenty-five animals had single ulcerated skin lesions on the nose and nine had nasal mucosa involvement (three of which also had a skin lesion). However, more recently, Mascarenhas et al. [102] reported the finding of advanced sporotrichosis in 15 dogs. These animals showed generalized cutaneous ulcers and crust, nodular and ulcerated lymphangitis on the hind limbs and one dog with generalized cutaneous ulcers, nasal and ocular involvement, exhibiting respiratory signs (nasal discharge, sneezing, stertorous breathing), anorexia and weight loss—signs of disseminated disease. All the animals studied showed positivity for *S. schenckii* in the culture of material from the lesions, which is curious, since *S. brasiliensis* is more associated with atypical and more severe forms of the disease [37]. Fungal elements are usually scarce and detected in lesions characterized by suppurative granulomatous inflammation [102].

### 3.4. Sporotrichosis Treatment

Spontaneous cure of sporotrichosis is rare and usually requires drug therapy with potassium iodide (POS), terbinafine (TRB) and Itraconazole (ITZ) that were administered orally, and Amphotericin B (AMB) deoxycholate or liposomal amphotericin B until clinical improvement, administered intravenously as summarized in Table 4.

In general, potassium iodide was used in the treatment of *S. schenckii* [73,74]. With the molecular identification of new species of the *Sporothrix* complex and, mainly, due to the emergence of *S*. *brasiliensis*, itraconazole became the drug of choice. The first studies on the subject pointed to its use at a dosage of 100 mg/kg/day [82,91], although, probably, due to continuous exposure to fungal spores in endemic areas, this dosage has increased [74]. At present, doses between 250 and 400 mg/kg/day have been recommended for the treatment of *S. brasiliensis* [110].

Most fungal susceptibility in vitro studies are based on the recommendations of the Clinical and Laboratory Standards Institute (CLSI). Prior to molecular identification methodologies, the CLSI document M38-A2 (2008) recommended the broth microdilution technique, starting from the conidia of the filamentous form of *S. schenckii* only [119]. In addition, interpretative minimal inhibitory concentration (MIC)/minimal effective concentration (MEC) or epidemiological cutoff values were not established for *Sporothrix* species. However, in 2017, a multicenter study [120] evaluated MIC/MEC of nine antifungals (AMB, five triazoles, TRB, flucytosine and caspofungin) for *S. schenckii* sensu stricto (301 strains), *S. brasiliensis* (486 strains), *S. globosa* (75 strains) and *S. mexicana* (13 strains). These assays were carried out by 17 laboratories located in Australia, Europe, India, South Africa and South and North America, and the ECVs were proposed for AMB (4 and 4 µg/mL), ITZ (2 and 2 µg/mL), voriconazole (64 and 32 µg/mL), KET and TRB (2 and 0.12 µg/mL) for *S. schenckii* and *S. brasiliensis*, respectively. In Brazil, ECV values for *S. brasiliensis* were proposed by Almeida-Paes [65]. The proposed ECV values were: 4.0, 2.0, 1.0, 2.0 and 0.25 for AMB, ITZ, KET, POS (posoconazole) and TRB, respectively [65]. A previous study [91], using the CLSI technique to determine the sensitivity of 46 canine isolates of *S. brasiliensis* and 1 isolate of *S. schenckii* reported that AMB presented the highest MIC values, and the other drugs (ITZ, KET, posaconazole and TRB) showed effective in vitro antifungal activity. The broth microdilution (M38-A2) in vitro antifungal susceptibility method was used by Valeriano et al. [93], Viana et al. [99], Oliveira et al. [116] and Ottonelli Stopiglia et al. [118] to assess the susceptibility of the *S. schenckii* complex from human, feline and canine clinical specimens.

Based on the ECV values, resistance as well as the effectiveness of therapy against *Sporothrix* can be monitored [65]. Mechanisms involved in the resistance of the *Sporothrix* complex to antifungals include: (i) the ability to produce melanin, (ii) the high polymorphism in size and number of chromosomes, and (iii) mutations in cytochrome P450 monooxygenases, particularly P450 CYP51, which is involved in ergosterol biosynthesis [62]. The emergence of failure in feline treatments reports and the increase in the number of strains displaying low susceptibility or resistance to ITZ stimulated the search for new drugs for the sporotrichosis treatment. Efforts have been made by different research groups worldwide, although with an emphasis on Brazilian groups (Table 5).

Other strategies such as local hyperthermia, achieved with the aid of a thermal bag, caused the regression of cutaneous lesions of feline sporotrichosis [130]. This technique has also been used to treat pregnant women and patients who have fixed cutaneous or lymphocutaneous lesions and who are intolerant to imidazole compounds, terbinafine hydrochloride, or iodine compounds. Cryosurgery, although already used to treat other mycoses, was also a tested technique for the treatment of sporotrichosis. Fichman et al. [131] tested this technique in four pregnant women between 16 and 24 weeks of pregnancy, aged between 18 and 32 years and who had contact or injuries caused by cats in the Rio de Janeiro city endemic area. Lesions were treated with two cycles of 10 to 30 s of freezing time. Treatment was monthly until the lesion healed. In 50% of cases, the causal agent was *S. brasiliensis*, and the clinical form of the lesions was classified as ulcer vegetative or nodular ulcer vegetative. Cryosurgery causes an increase in antigen-presenting dendritic cells (DCs), neutrophils and macrophages in subcutaneous tissue, as well as migration of DCs to regional lymph nodes [132]. This technique has been shown to be an alternative to the treatment of sporotrichosis in pregnant women [131].

Nonetheless, the provision of public veterinary services and free antifungal drug supplies is restricted and cannot meet the demand for veterinary medical care for all cases. Treatment abandonment occurs with high frequency in cases of feline sporotrichosis due to the longevity (6 to 12 months) and high costs of antifungal treatment, difficulty in oral administration of drugs to infected animals, representing an obstacle to the control of this disease [133]. Besides, factors such as the low availability of antifungal drugs for the treatment of sporotrichosis cases in its various clinical forms, the high cost of maintaining patients who progressed to disseminated or atypical forms in intensive care units in the Unified Health System (SUS) and the social inequalities, which impose precarious livelihoods for the Brazilian population most affected by the disease, contribute to the spread of feline and zoonotic sporotrichosis [35,112].

### 3.5. Prophylaxis and Prevention

Controlling the source of sporotrichosis infection and identifying the factors involved in the dynamics of zoonosis transmission contribute to the adoption of surveillance and control measures necessary to contain the disease. Some simple measures like contact with domestic cats, especially in endemic areas, should be avoided since there are reports of acquiring sporotrichosis after contact even without biting or scratching [32,112]. Neutering pet cats should also be considered as it makes cats more homely and less likely to walk around, reducing exposure to animals or contaminated environments [17,30,47]. Furthermore, when a case is detected, it must remain in quarantine, indoors and away from other cats to prevent transmission. The entire environment must be disinfected with 1% sodium hypochlorite solution or 70% alcohol. Quilts, cloths or similar used by the animal to sleep, play or rest should be discarded [23]. If necessary, treat the environment with antifungal sprays. However, if the animal dies, it cannot be buried, as this would spread the fungus in the environment. The body needs to be cremated. However, in most cases, even with the tutor present, the animal is mistakenly abandoned to die on the street [96]. On the other hand, homeless animals end up dying and being left outdoors. These situations contribute to the disease persistence and allow the fungus to spread and reproduce in the environment, affecting other cats abandoned even though healthy, who are homeless. In addition, a previous study showed that feces from sick animals can contaminate the soil with fungus propagules and contribute to the spread and persistence of the fungus in the environment [73].

The use of appropriate clothing and gloves while working in the field is also suitable for prevention [82]. In the case of veterinarians, an N95 mask must be used to prevent the entry of spores into the respiratory tract, eye protection, a long-sleeved apron and the use of examination gloves, essential when handling patients who present symptoms characteristic of sporotrichosis [56,57]. Furthermore, doctors and veterinarians must notify the state or local public health department if they detect cases in humans or animals [82].

Early diagnosis is still the best therapy, considering that both in humans and cats, treatment is long and expensive, which often leads to the animal being abandoned to die on the streets, spreading the disease to stray cats, albeit healthy ones. In this context, the public provision of adequate medication for the treatment of low-income patients and sick animals would be essential to minimize the spread of the disease [99] as well as the incentive to innovation and production of new drugs, diagnostic methods and vaccines.

## 4. Conclusions

The sporotrichosis has become an important public health problem in Latin American countries, particularly in Brazil, where it is still considered neglected [134]. This disease has contributed to the maintenance of inequality in the country’s development and permanence of the poverty conditions of the affected individuals [96]. Thus, the implementation of prevention and control strategies in joint actions with epidemiological surveillance, laboratory, environmental and animal is necessary and urgent. The implementing of public policies that define roles and responsibilities of governmental and non-governmental managers related to the environment, health, and animal welfare as well as health education practices for professionals and the general population of endemic countries can contain the sporotrichosis epidemic and break the cat–human–environment transmission. However, for this, it is important to include the sporotrichosis as a neglected disease in the list of neglected tropical diseases of the OPAS and OMS. This inclusion will be possible only through collective work by research institutes and health centers, and assistance for humans and animals with sporotrichosis and implementation of sporotrichosis in compulsory notification in all of Brazil and Latin America.

Nonetheless, in Brazil, there is a lack of structuring and training of people who work in the network of public health laboratories in the identification of fungi. In addition, there is a lack of availability of laboratory supplies to carry out the mycological diagnosis, as well as precariousness in the techniques of identification of agents associated with cases of sporotrichosis, especially in animals, in most Brazilian states. Access to diagnosis and the number of cases are important factors that go together and affect decision-making by health entities. Sporotrichosis is a neglected mycosis since 1971 as reported by Orr and Riley [135].

## Figures and Tables

**Table 1:** Study inclusion and exclusion criteria adopted for the selection of studies.

Inclusion Criteria	Exclusion Criteria
Narrative and systematic reviews	Editorials
Original articles	Opinion papers
Cross-sectional or longitudinal studies	Thesis/Dissertation
	Meeting Summary
Studies written in English, Spanish or Portuguese	Book chapters
Studies from developed and developing countries	

**Table 2:** Classical diagnostic methods for sporotrichosis in the analysis of clinical specimens (pus, secretions, biopsies).

Specimens	Direct Exam (KOH)	Cytological Examination (Grocott/PAS/Gram/Giemsa)	Fungal Culture	Reference
Humans	Low diagnostic sensitivity. Difficult direct examination	Low fungal load in the lesions. Difficult direct examinations	Reference standard. Melanin production.	[45],^58^,^59^,^60^,^61^]
Felines	High fungal load favoring the direct examination	Cytologic examination—preliminary diagnosis. High sensitivity (79 to 85%)	Reference standard. Similar sensitivity to skin biopsy. Melanin production related to azole resistance.	[46],^61^,^62^,^63^]
Canines	Low sensitive for the diagnosis Difficult direct examination	Low fungal load in the lesions. Low sensitivity (32%)	Reference standard. Fungal load is usually low.	[61]

**Table 3:** Immunological (serodiagnostic), molecular, spectrometric and combination diagnostic methods used for the diagnosis of sporotrichosis.

Methods	Patient Type	Species Detected	Limitation	References
ELISA (Enzyme Linked ImmunonoSorbent Assay)	Human, Feline	*S. schenckii* *S. brasiliensis*	Cross-reactions with other fungal diseases may occur. Addition of 6M urea reduces cross-reactivity. Decrease in serum antibody titers as the lesions heal. Combine with clinical specimen cultures.	[64,65,66,67]
Molecular diagnostic methods and gene sequencing	Human, Feline, Canine	*S. brasiliensis, S. schenckii, S. globosa, S. mexicana, S. pallida*	High cost	[9,10,11], [13,14,15], [20,21], [32], [52,53], [56], [68]
MALDI-TOF-MS (Matrix Assisted Laser Desorption Ionization Time-of-Flight Mass Spectrometry)	Human, Feline, Canine	*S. brasiliensis, S. schenckii, S. globosa, S. pallida, S. mexicana, S. luriei*	High cost	[69,70]
Histological methods associated to PCR-based molecular diagnostic	Feline	*S. chilensis, S. mexicana, S. pallida, S. globosa, S. brasiliensis, S. schenckii*	Use of formalin-fixed and paraffin-embedded tissues	[71,72]

**Table 4:** Antifungal drugs used for the treatment of sporotrichosis.

Antifungal	Administration Route	Patients	Dosage	Indications	Reference
Potassium iodide (KI)	oral	Humans (children/elderly)	15 mg/kg/day	Humans in the endemic area	[74], [76], [81,82], [87], [89,90], [96]
		cats	2.5–20 mg/kg/day plus ITZ cat dosage	Cats presenting multiple skin and mucosal lesions, or presence of respiratory signs; Cases refractory to ITZ monotherapy	
Itraconazole (ITZ)	oral	Humans (adults)	100 to 400 mg/day	Healthy patients with limited lesions, immunosuppressed patients and in the systemic form	[74], [77,78,79], [82], [87], [90,91,92,93], [96], [105,106,107,108,109,110,111,112,113,114,115,116,117,118]
		cats	25 mg–100 mg/kg/day	Cats with fixed cutaneous lesions and naïve to antifungal therapy	
Terbinafine (TRB)	Oral	Humans (adults)	250–500 mg/day	Cutaneous sporotrichosis; cases which itraconazole was contraindicated	[87], [91], [99], [107], [115], [118]
		cats	30 mg/kg/day		
Amphotericin B (AMB)	Deoxycholate (intravenous)	Humans (adults)	0.3–1 mg/kg/day	Disseminated forms sporotrichosis	[80], [84], [86], [87], [91], [93], [96], [107], [117,118]
	Liposomal (intravenous)	Humans (adults) and cats (rarely)	3–5 mg/kg/day		

**Table 5:** New antifungal drugs for potential use in the treatment of sporotrichosis.

Antifungal Drug	Action Mechanism	Efficiency	Reference
22-hydrazone-imidazolin-2-yl-chol-5-ene-3β-ol (H3)	inhibition of Δ (24)-sterol methyltransferase	*Sporothrix brasiliensis; Sporothrix schenckii*	[121]
CTZ, KTZ and their respective metal salts or metal complexes under mild conditions	interference with the cell shape	*S. schenckii, S. brasiliensis, Sporothrix globosa*	[122]
Tacrolimus and cyclosporine A alone and in combination with ITZ or FLZ	inhibiting of calcineurin (only tacrolimus showed synergism with azoles)	*S. brasiliensis, S. schenckii*	[123]
Miltefosine	changes in the lipid composition of membranes	*S. brasiliensis* with low susceptibility to AMB or ITZ	[124]
Structural analogues of miltefosine (TCAN26, TC19, and TC70)	disruption of the cell membrane and cell wall, and increased cell wall thickness	*S. schenckii sensu stricto*	[125]
1,4-naphthoquinone derivatives	not described	*S. schenckii*	[126]
2,3-disubstituted-1,4-naphthoquinones	not described	*S. schenckii*	[127]
Substituted α- and β-2,3-dihydrofuranaphthoquinones	arylation of the thiol groups of proteins, intercalation, induction of breaks in the DNA chain, generation of free radicals and other reactive oxygen species (ROS), and bioreductive alkylation via the formation of quinone methide	*S. brasiliensis, S. schenckii*	[128]
Pathogen Box library (400 compounds evaluated; 12 showed effectiveness)	disrupted cells and overflow of intracellular content, increase in cell size, accumulation of neutral lipids, and disruption of plasma membrane integrity	*S. brasiliensis, S. schenckii*	[129]

CTZ = clotrimazole; KTZ = ketoconazole; ITZ = itraconazole; FLZ = fluconazole; AMB = amphotericin B.

## Data Availability

Not applicable.
